# Mesoporous Silica Nanoparticles Coated with Carboxymethyl Chitosan for 5-Fluorouracil Ocular Delivery: Characterization, In Vitro and In Vivo Studies

**DOI:** 10.3390/molecules28031260

**Published:** 2023-01-27

**Authors:** Adel Ali Alhowyan, Mohd Abul Kalam, Muzaffar Iqbal, Mohammad Raish, Ahmed M. El-Toni, Musaed Alkholief, Aliyah A. Almomen, Aws Alshamsan

**Affiliations:** 1 Department of Pharmaceutics, College of Pharmacy, King Saud University, Riyadh 11451, Saudi Arabia; 2 Nanobiotechnology Unit, College of Pharmacy, King Saud University, Riyadh 11451, Saudi Arabia; 3 Department of Pharmaceutical Chemistry, College of Pharmacy, King Saud University, Riyadh 11495, Saudi Arabia; 4 Central Lab, College of Pharmacy, King Saud University, Riyadh 11451, Saudi Arabia; 5 King Abdullah Institute for Nanotechnology, King Saud University, Riyadh 11495, Saudi Arabia; 6 Nanomaterials and Nanotechnology Department, Central Metallurgical Research and Development Institute (CMRDI), Helwan, Cairo 11865, Egypt

**Keywords:** 5-fluorouracil, mesoporous silica, nanoparticle, ocular delivery, corneal permeability study, ocular pharmacokinetic

## Abstract

This study investigates the development of topically applied non-invasive amino-functionalized silica nanoparticles (AMSN) and O-Carboxymethyl chitosan-coated AMSN (AMSN-CMC) for ocular delivery of 5-Fluorouracil (5-FU). Particle characterization was performed by the DLS technique (Zeta-Sizer), and structural morphology was examined by SEM and TEM. The drug encapsulation and loading were determined by the indirect method using HPLC. Physicochemical characterizations were performed by NMR, TGA, FTIR, and PXRD. In vitro release was conducted through a dialysis membrane in PBS (pH 7.4) using modified Vertical Franz diffusion cells. The mucoadhesion ability of the prepared nanoparticles was tested using the particle method by evaluating the change in zeta potential. The transcorneal permeabilities of 5-FU from AMNS-FU and AMSN-CMC-FU gel formulations were estimated through excised goat cornea and compared to that of 5-FU gel formulation. Eye irritation and ocular pharmacokinetic studies from gel formulations were evaluated in rabbit eyes. The optimum formulation of AMSN-CMC-FU was found to be nanoparticles with a particle size of 249.4 nm with a polydispersity of 0.429, encapsulation efficiency of 25.8 ± 5.8%, and drug loading capacity of 5.2 ± 1.2%. NMR spectra confirmed the coating of AMSN with the CMC layer. In addition, TGA, FTIR, and PXRD confirmed the drug loading inside the AMSN-CMC. Release profiles showed 100% of the drug was released from the 5-FU gel within 4 h, while AMSN-FU gel released 20.8% of the drug and AMSN-CMC-FU gel released around 55.6% after 4 h. AMSN-CMC-FU initially exhibited a 2.45-fold increase in transcorneal flux and apparent permeation of 5-FU compared to 5-FU gel, indicating a better corneal permeation. Higher bioavailability of AMSN-FU and AMSN-CMC-FU gel formulations was found compared to 5-FU gel in the ocular pharmacokinetic study with superior pharmacokinetics parameters of AMSN-CMC-FU gel. AMSN-CMC-FU showed 1.52- and 6.14-fold higher AUC0-inf in comparison to AMSN-FU and 5-FU gel, respectively. AMSN-CMC-FU gel and AMSN-FU gel were “minimally irritating” to rabbit eyes but showed minimal eye irritation potency in comparison to the 5 FU gel. Thus, the 5-FU loaded in AMSN-CMC gel could be used as a topical formulation for the treatment of ocular cancer.

## 1. Introduction

5-Fluorouracil (5-FU) is commonly used to treat many cancer diseases including epithelial cancers. The ocular solution of 5-FU can be used for conjunctival/corneal squamous cell carcinoma [1]. The treatment regime for corneal and conjunctival intraepithelial neoplasia, squamous cell carcinoma, and malignant melanoma of conjunctiva can be a pulse ocular doses of 1% 5-FU four times a day for 4 days, for several treatment cycles at monthly intervals. However, the lesion may recur and retreatment is required with an excisional biopsy. Long-term follow-up is necessary for these patients as the lesions may recur after the therapy [1,2,3]. Usually, 5-FU eye drops are prepared as an extemporaneous preparation and the solution would be stable for only 7 days [4].

Particles with a mucoadhesive property have been extensively investigated as carriers of ocular drug delivery [5,6]. Mucoadhesive particles can be better attached to the mucin on the ocular surface, which increases the residence time in the preocular region. Increasing the residence time of the particles and slowly releasing the loaded drug would improve the ocular bioavailability of the drug. By this approach, many researchers tried nano- or micro-particles made of mucoadhesive polymers such as poly (ethylene glycol), poly (acrylic acid), chitosan, and sodium carboxymethylcellulose for ocular drug delivery. Mucoadhesive materials need to possess a functional group that forms a bond with the mucin. Kim et al. (2018) proposed an amino-functionalized mesoporous silica nanoparticle (AMSN) for the ocular delivery of brimonidine for glaucoma. As AMSN has two functional groups, hydroxyl and amine groups, the nanoparticles would be mucoadhesive. The hydroxyl groups could form hydrogen bonds with the mucin and the positive charge of the amino groups in the AMSN could also form an ionic complex with the negatively charged groups in mucin. Furthermore, the drug-loaded AMSN could be released in a controlled manner from the particles to maintain a concentration gradient, which improves the drug permeation through the corneal barriers. Kim and coworkers suggested that loaded AMSN with brimonidine can increase the ocular bioavailability of the drug by increasing the residence time at the preocular surface and controlling the release of the drug [7].

Chitosan polymers such as carboxymethyl chitosan (CMC) are suitable for coating nanoparticles by electrostatic interaction and can enhance the physicochemical properties of nanoparticles. Several chitosan-containing products were approved by the US Food and Drug Administration. Chitosan has antibacterial activity and mucoadhesive properties, and it can improve the ocular penetration of some drugs by acting as a penetration enhancer [8,9,10,11]. In addition, the CMC derivative, which is an amphoteric chitosan derivative, has better physicochemical properties than chitosan such as better solubility, better mucoadhesion, and antibacterial activity [8]. Moreover, CMC was proposed as a material with good potential to prolong the corneal residence time of drugs, and CMC-coated nanoparticles were more efficient in enhancing the intraocular penetration of some drugs [8]. Based on these considerations, the hypothesis is that 5-FU loaded in AMSN and CMC-coated AMSN would increase the residence time of 5-FU at the precorneal and enhance the permeation of the drug inside the ocular structures [7,8,10,11,12].

Topical ophthalmic drops are the first choice of the physician due to patient compliance. Despite their simplicity, eye drops present some problems such as low ocular drug bioavailability, pulse drug entry after topical administration, and repeated dosing giving rise to ocular irritation [9]. The side effects of 1% 5-FU eye drops would occur more frequently because this is considered a high concentration, hence causing high systemic exposure to the chemotherapeutic agent. 5-FU suffers from multiple shortcomings that limit its clinical applications such as short half-life, disease resistance, non-specific in vivo distribution, and severe adverse reactions [13]. The side effects of topical 5-FU include redness and pain at the instillation side, eyelid swelling, filamentary keratitis, conjunctival congestion, epitheliopathy, and, rarely, superficial stromal melting. Canalicular stenosis may occur with systemic 5-FU administration but not with topical 5-FU. To reduce the side effects, usually topical corticosteroids are used along with preservative-free artificial tears [14]. Similarly, 5-FU is a highly polar molecule with a log *P* value of −0.89, which decreases its biological membrane permeability [13]. This problem encouraged the development of a suitable carrier loaded with 5-FU to attain high drug bioavailability at lower doses, reducing the dosing frequency, and minimizing the systemic drug absorption [9]. The objective of this work was to develop 5-FU-loaded AMSN and CMC-coated AMSN loaded with 5-FU for ocular delivery with better bioavailability.

## 2. Results and Discussion

### 2.1. Formulation Development Encapsulation and Loading Efficiencies

Synthesis and characterization of AMSN-FU were previously described in detail in our previous research [15]. AMSN-CMC-FU was prepared by two different methods. The AMSN samples with first loaded with 5-FU and then coated with CMC (F1) or first coated with CMC and then loaded with 5-FU (F2). The EE% and LC% were found higher for F2 than F1. The EE% and LC% for F2 were 25.8 ± 5.8% and 5.16 ± 1.2%, respectively. The EE% and LC% of F1 were found 10.33 ± 3.25 and 1.94 ± 0.61, respectively (Table 1). This is could be due to the displacement of CMC for 5-FU molecules in F1. In the F2 formulation, a part of the drug was entrapped in AMSN and another part in the surface polymer layers. F2 (AMSN-CMC-FU) was chosen for further studies due to the higher EE% and LC% in comparison to F1.

### 2.2. Particle Morphology by SEM and TEM

**Figure 1:**
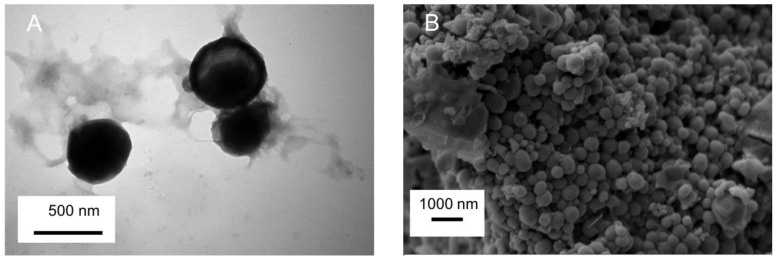
The TEM image of AMSN-CMC-FU (**A**) and the SEM image of AMSN-CMC-FU (**B**).

TEM images of AMSN-CMC-FU (F2) are shown in Figure 1A. In previous work, we showed that the structure of AMSN was well ordered mesoporous structure with an average particle size of 90–230 nm [15]. The TEM photograph of AMSN indicates an almost uniform nature and spherical shape. In image A, the surface of AMSN-CMC-FU shows the successful coating of polyelectrolyte and the disappearance of the mesopore of the AMSN and the nanoparticle retains the spherical shape with increasing size. The SEM micrograph (Figure 1B) of AMSN-CMC-FU suggested larger particles compared to AMSN due to the coating of CMC on the AMSN. The DLS analysis of AMSN-CMC-FU found a particle size of 249.4 nm with a polydispersity of 0.429 for AMSN-CMC-FU. Similar to other researchers, our previous work showed that nanoparticles of around 200 nm can improve the ocular permeation of some drugs [4,5,6]. We do not expect that all nanoparticles in these formulations permeate the corneal surfaces as only very small particles permeate this membrane. However, AMSN-FU and AMSN-CMC-FU would allow 5-FU to permeate by maintaining the drug concentration gradient across the membrane by controlling the release of the drug and increasing the drug contact/residence time with tissue.

### 2.3. Zeta Potential

The zeta potential study helps to confirm the CMC deposition on the AMSN assembly and the final charge of AMSN-CMC. The values of the zeta potential of AMSN possess a positive potential of 6.36 ± 1.49 and the loaded AMSN (AMSN-FU) has a zeta potential of +30.4 ± 5.71 mV in an aqueous solution. Conversely, the zeta potential for AMSN-CMC and loaded AMSN-CMC-FU were −29.9 ± 4 mV and −5.72 ± 4.04 mV, respectively with negative surface charge suggesting the successful coating of the AMSN with CMC. The stability and ocular permeation of the nanoparticles would be improved if we used the formulations in a gel form. We theorized that the nanoparticle gel formulation would increase the residence and contact time of the system with the ocular surface, and interact with mucin, hence allowing more drugs to diffuse through the corneal tissues. AMSN, Carbopol gel, and Chitosan polymers were identified as mucoadhesive, and increase the bioavailability of drugs in ocular application [1,4,7]. The superiority of the CMC gel formulation would be due to the higher mucoadhesion strength as well as the opening of the tight junction of the epithelial layer of the cornea [1,4]. CMC possesses two functional groups (carboxylic and amino groups) that would form ionic interactions and hydrogen bonds with mucin, which increase the mucoadhesion strength of the formulation [1]. AMSN would also exert a mucoadhesion but this would be lower than that of the coated formulation with CMC.

### 2.4. Proton Nuclear Magnetic Resonance (^1^H-NMR) Spectroscopy

**Figure 2:**
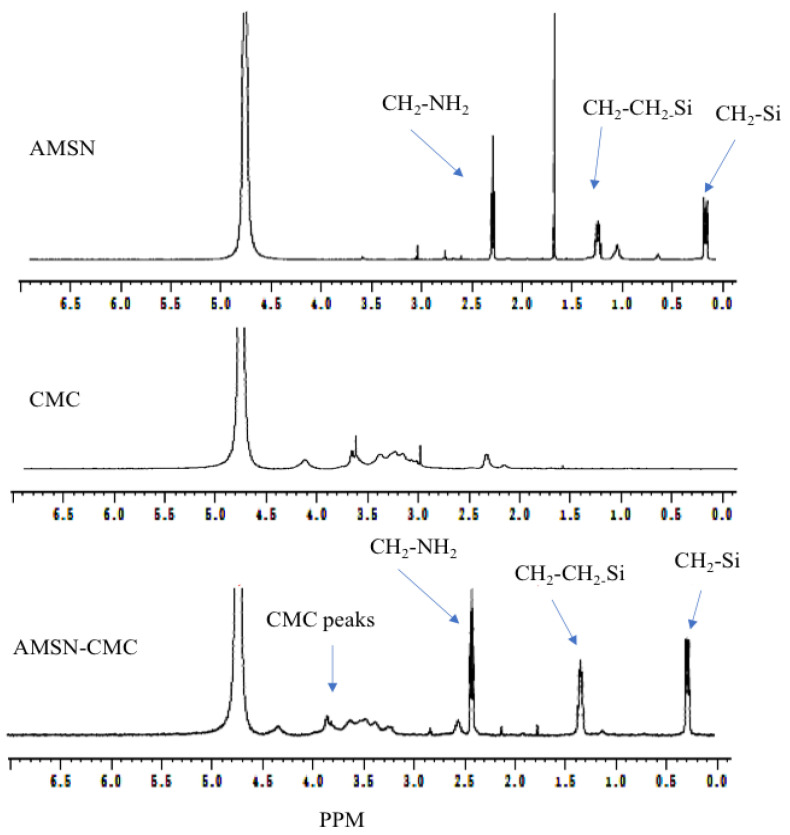
Proton NMR spectra of AMSN, CMC, and AMSN-CMC.

**Figure 3:**
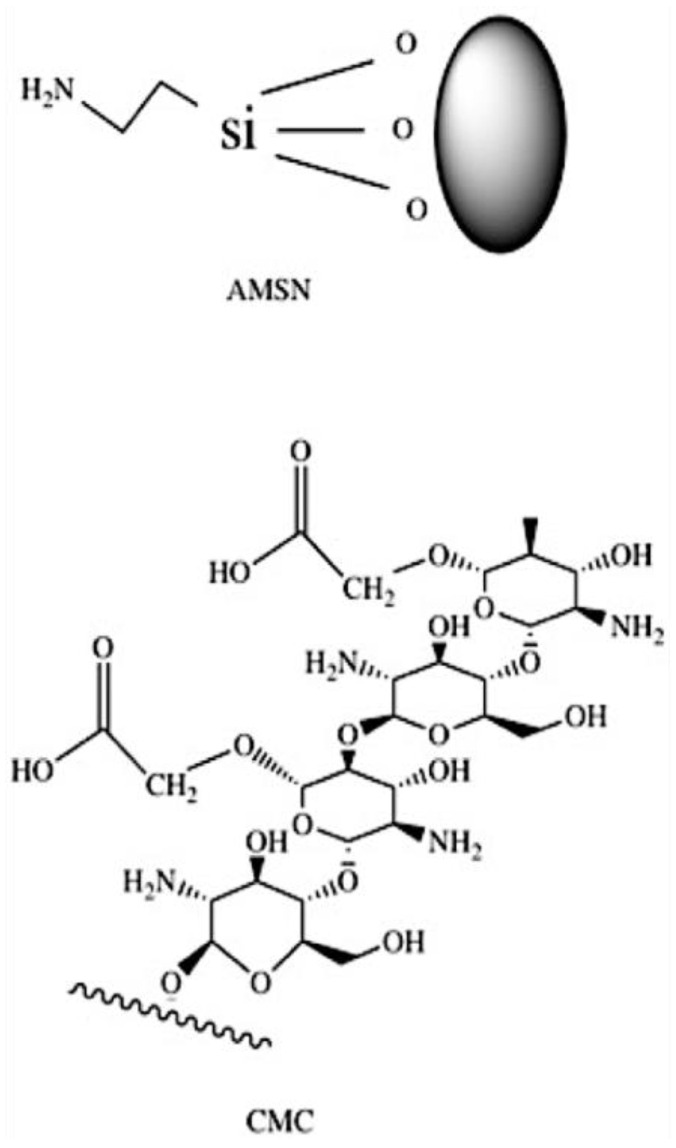
Chemical structure of AMSN and CMC.

The ^1^H-NMR spectra of AMSN, CMC, and coated AMSN-CMC are presented in Figure 2, while the chemical structures of AMSN and CMC are illustrated in Figure 3. The freeze-dried samples were solubilized in sodium hydroxide and measured in D_2_O because the spectra were not clear when measured directly in D_2_O. The spectrum of AMSN was in good agreement with mesoporous silica nanoparticles functionalized with APTES in an alkaline medium found in the literature [16,17,18,19]. The three methylene groups of the 3-aminopropyl group appear at 2.26 (CH_2_ connected to N), 1.2 (middle CH_2_), and 0.1 ppm (CH_2_ connected to Si), respectively. The hydrogen peaks were shifted to lower ppm due to the measurement of the sample in an alkaline media [16]. The strong peak at 2.2 ppm could be due to impurities in the sample. These peaks further confirmed the successful preparation of AMSN illustrated in the previous work [15]. The NMR spectrum of CMC showed a peak at 2.4 ppm, which could be related to the CH_3_ in the acetamide groups. The small peak at 4.17 ppm could be related to the carboxymethyl protons (-O-CH_2_-COOD) in the CMC [20,21,22]. In addition, all distinctive peaks of CMC were retained in good agreement with the NMR spectrum of this polymer found in the literature [20,21,22]. Moreover, all peaks of AMSN and CMC were retained in the AMSN-CMC spectrum suggesting the successful coating of AMSN with CMC.

### 2.5. Thermogravimetric Analysis (TGA)

**Figure 4:**
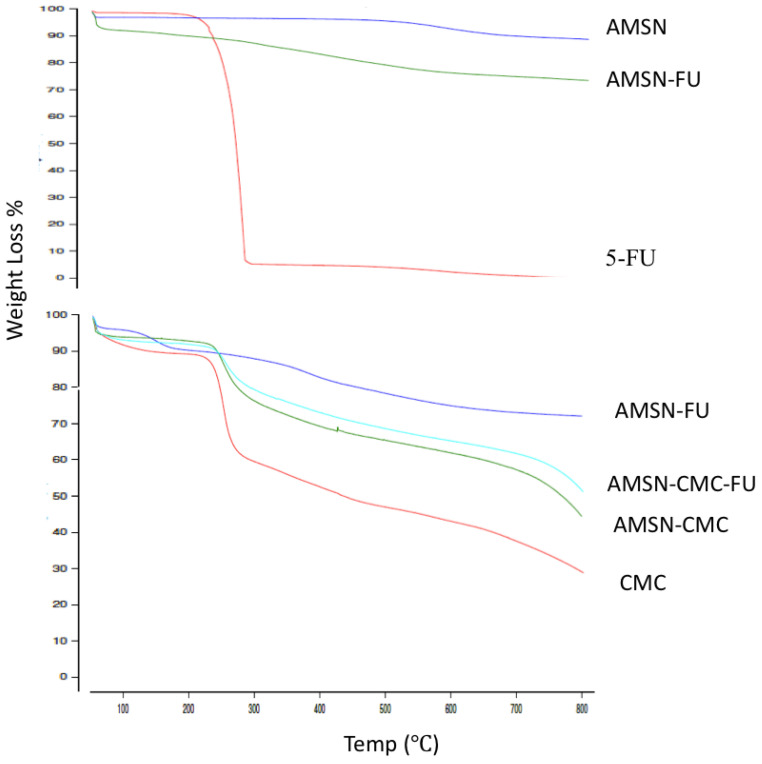
TGA thermograms of (upper figure) 5-FU, AMSN, and AMSN-FU; (lower figure) CMC, AMSN-CMC, AMSN-FU and AMSN-CMC-FU.

Differences in the thermograms were apparent between the AMSN and AMSN-FU (Figure 4). TGA analysis was carried out to calculate the overall amount of 5-FU loaded into AMSN (Figure 4). The obtained values were in good agreement with the drug loading measured by indirect methods using HPLC (around 15%). The reference thermograms of pure 5-FU and pure AMSN were measured for comparison. The thermogram of 5-FU was characterized by a considerable weight loss at 276 °C due to the drug decomposition process. The thermogram of pure AMSN showed low weight loss in the temperature range of 40–800 °C, which suggested the high thermostability of AMSN. The weight loss of AMSN was around 11% and the difference in weight loss between AMSN and loaded AMSN-FU was considered to arise from the amount of the drug loaded inside the nanoparticles [23,24]. The thermograms of AMSN-CMC and AMSN-CMC-FU showed a large weight loss due to the loss of CMC from the surface of AMSN. The mass ratio of the coated CMC layer on the surfaces of AMSN was around 45% for AMSN-CMC and around 40% for AMSN-CMC-FU. These thermograms confirmed the successful coating of AMSN with CMC. In addition, AMSN-CMC-FU showed slightly lower weight loss than drug-free AMSN-CMC due to the replacement of a part of the high molecular weight CMC polymer with 5-FU [7,25].

### 2.6. Powder X-ray Diffraction Patterns (PXRD)

**Figure 5:**
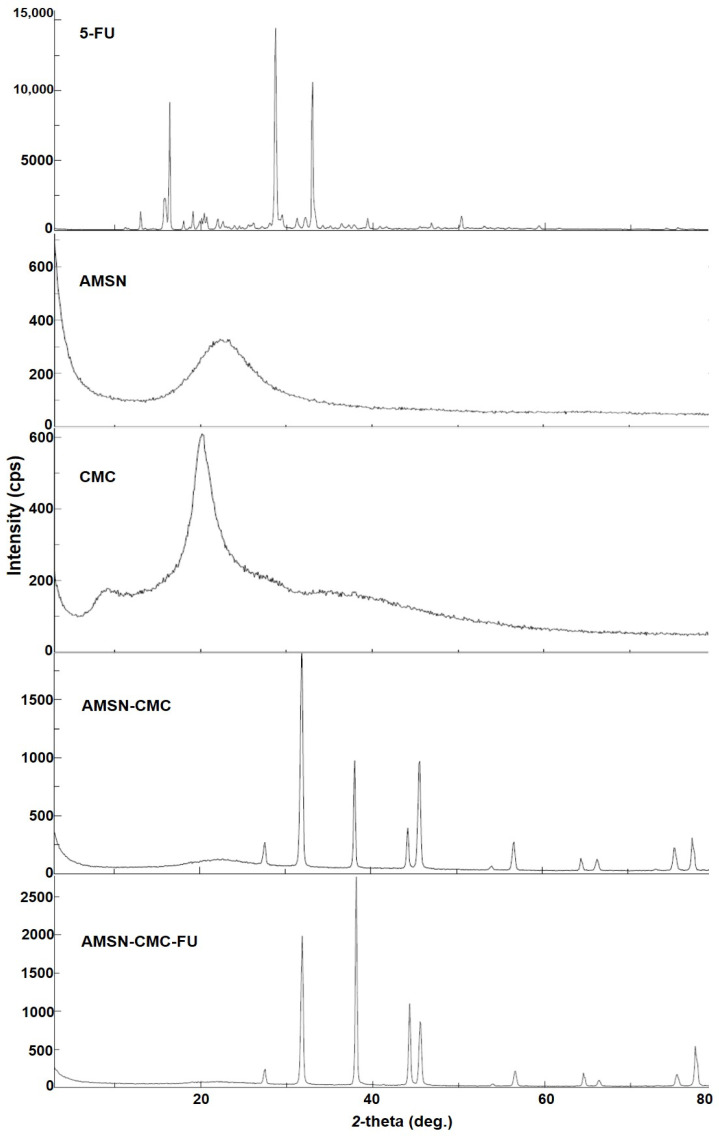
Powder X-ray diffraction patterns (PXRD) of 5-FU, AMSN, CMC, AMSN-CMC, and AMSN-CMC-FU.

The PXRD patterns of 5-FU, AMSN, CMC, AMSN-CMC, and AMSN-CMC-FU were recorded to determine the possible differences in the crystalline nature of the prepared materials (Figure 5). The appearance of the broad peak with a *2θ* value ranging from 17 to 25° in the PXRD pattern of AMSN suggests its amorphous nature. The diffractogram of AMSN-CMC showed intense peaks from 30 to 80°, suggesting more crystalline structures of the polymer on the surface of AMSN. The diffraction pattern of AMSN-CMC-FU showed a similar pattern to that of AMSN-CMC with intense peaks from 30 to 80°, and the disappearance of 5-FU peaks, suggesting the successful encapsulation of 5-FU into the AMSN-CMC-FU and the coated CMC layer in the crystalline form [26].

### 2.7. Fourier Transform Infrared Spectroscopy (FTIR)

**Figure 6:**
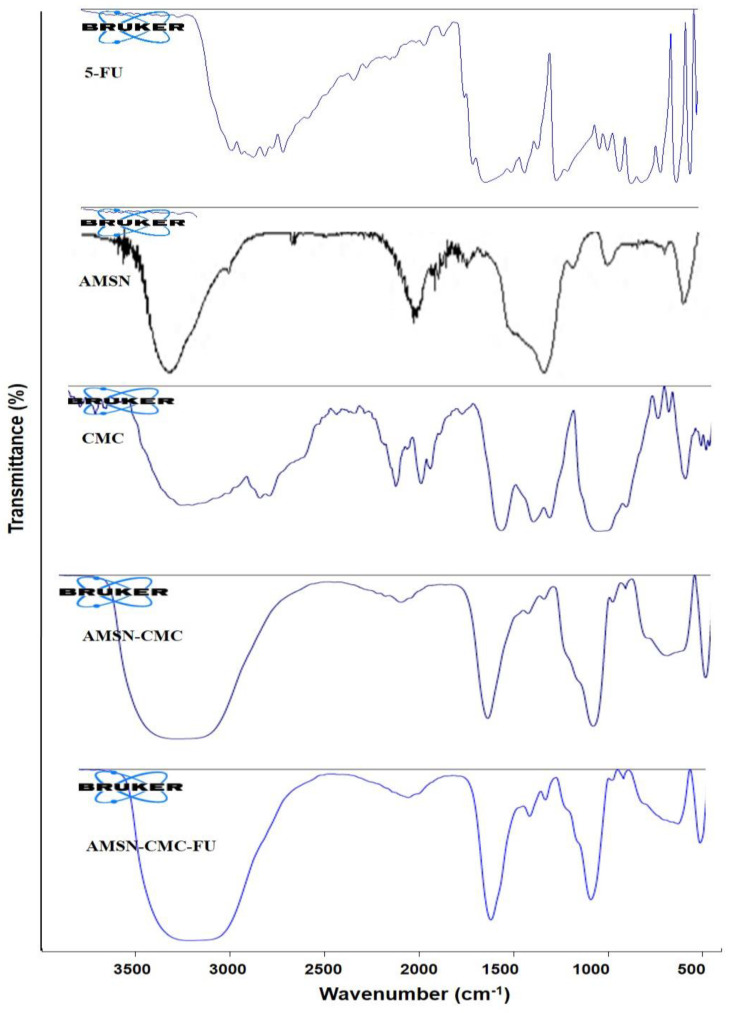
FTIR spectra of 5-FU, AMSN, CMC, AMSN−CMC, and AMSN−CMC−FU.

The FTIR spectra of 5-FU, AMSN, CMC, AMSN-CMC, and AMSN-CMC-FU are presented in Figure 6. The spectrum of AMSN showed a peak at 1046 cm^−1^, which was the peak of the Si-O-Si group. A peak observed at 1550 cm^−1^ and a broad peak between 2900 and 3400 cm^−1^ were assigned to N-H bending and stretching vibrations of primary amines, respectively. The peak at 1468 cm^−1^ could be due to the bending vibrations of either ammonium ion N-H bonds or the methylene C-H bonds. The CMC spectrum showed the characteristic peaks of CMC at 1599 and 1405 cm^−1^, indicating the presence of carboxy and carboxy methyl groups, respectively. Two peaks at 1082 and 1323 cm^−1^ were assigned to the C-O stretching band and a merging of the carboxymethyl group at the OH group was present in the C6 position of CMC. A broad peak around 3300 cm^−1^ indicated O-H stretching and intermolecular hydrogen bonds of the polysaccharide. In the spectrum of AMSN-CMC, the 1049 cm^−1^ peak could be assigned to Si-O-Si stretching. The band retained at 3300 cm^−1^ indicated the effective bonding of CMC to AMSN. Additionally, a peak at 1635 cm^−1^ indicated free COOH groups on the surface. The strong peaks of 5-FU disappeared from the AMSN-CMC-FU spectrum, suggesting successful encapsulation of 5-FU. Overall, the FTIR results confirmed the successful surface functionalization of AMSN by CMC and encapsulation of 5-FU inside AMSN [15,26].

### 2.8. Evaluation of the Mucoadhesive Strength Using Mucin (Particle Method)

**Figure 7:**
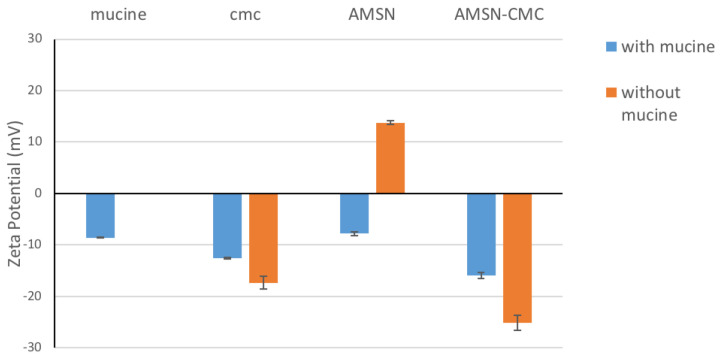
Change in the zeta potential of AMSN and AMSN-CMC nanoparticles after incubation with mucin.

This method can be used to measure the ability of the particle to interact with the mucin in the corneal epithelial cells, which in turn plays an important role in the formulation of mucoadhesion. Carbopol was verified for its efficacy as a mucoadhesive polymer in pharmaceutical formulation. Hoffmann and Daniels evaluated the mucoadhesion of fast-dissolving tablets containing Carbopol and they found that the formulations containing Carbopol showed up to three-fold more adhesion to mucosal surfaces than the formulation without Carbopol [27]. Thus, we used all formulations as a Carbopol gel. The mucoadhesive properties of the nanoparticles not impeded in the gel were investigated using the mucin particle method by measuring the change in the zeta potential of the mucin mixture after incubation with the nanoparticles. For the intact AMSN, the zeta potential was around +13.75 mV (Figure 7) and this positive surface charge is due to the amino groups in the AMSN. However, after incubation in the mucin solution for 48 h, the zeta potential shifted to −7.74 mV, which was closer to that of the mucin at −8.64 mV, implying the presence of mucin adsorbed on the AMSN surface [7,27,28]. Similar to other silica particles, there are many hydroxyl groups in the AMSN, allowing for the formation of a hydrogen bond with the mucin. The amino groups in the AMSN would adhere better to the mucin compared with mesoporous silica nanoparticles without amino groups, which could be due to the formation of an ionic complex with the negatively charged mucin [7]. Conversely, CMC and AMSN-CMC have a negative zeta potential of −17.37 mV and −25.133 mV, respectively. After incubation with mucin, the zeta potential of CMC and AMSN-CMC has shifted to a value closer to the mucin values −12.533 mV and −15.9 mV, respectively. Chitosan polymer is known to have mucoadhesion properties [7,29,30]. CMC is an amphoteric derivative of chitosan with two functional groups, the amino group and the carboxylic acid group, that would interact with mucin. Thus, CMC and nanoparticles coated with CMC are expected to expert higher mucoadhesion strength in comparison to other molecules with one functional group that could interact with mucin [8,31].

### 2.9. In Vitro Drug Release Study

**Figure 8:**
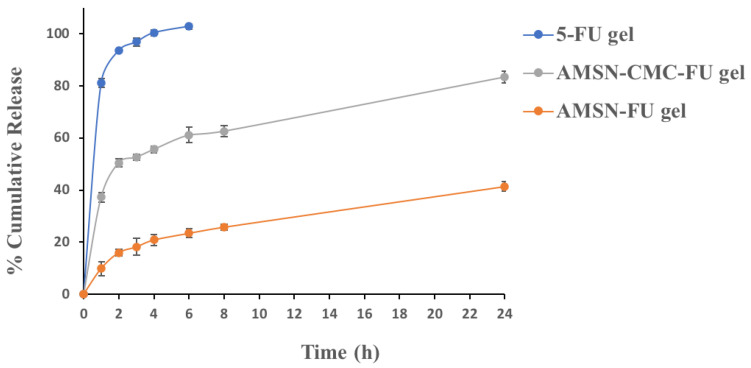
In vitro release of 5-FU gel, AMSN-FU gel, and AMSN-CMC-FU gel. The values are represented as the mean of three measurements with standard deviation (Mean ± SD, *n* = 3).

The in vitro release study of the drug from 5-FU gel, AMSN-FU gel, and AMSN-CMC-FU gel was performed in phosphate buffer saline (PBS, pH 7.4). The release profiles are presented in Figure 8. The drug release from AMSN-CMC-FU gel showed around 37.23% of 5-FU was released after 1 h, while the uncoated AMSN-FU gel showed about 9.87% after 1 h. After 24 h 83.38% and 41.41% of 5-FU were released from AMSN-CMC-FU gel and AMSN-FU gel, respectively. In the case of free 5-FU gel, 100% of 5-FU was released after 4 h with a high burst effect (81.23% in 1 h). The burst release of the drug from the nanoparticle formulations could be due to the amount of the drug adsorbed on the surface [7]. The higher burst release from AMSN-CMC-FU gel in comparison to AMSN-FU Carbopol gel could be due to the high amount of drug adsorbed on the CMC polymer layer as the drug loading was performed after the coating of AMSN with CMC [26]. The nanoparticle release profiles showed a more controlled release in comparison to free 5-FU gel, which could be beneficial for enhancing of permeability of the drug through the corneal surfaces by maintaining the concentration gradient [32].

### 2.10. Ex Vivo Corneal Permeation

**Figure 9:**
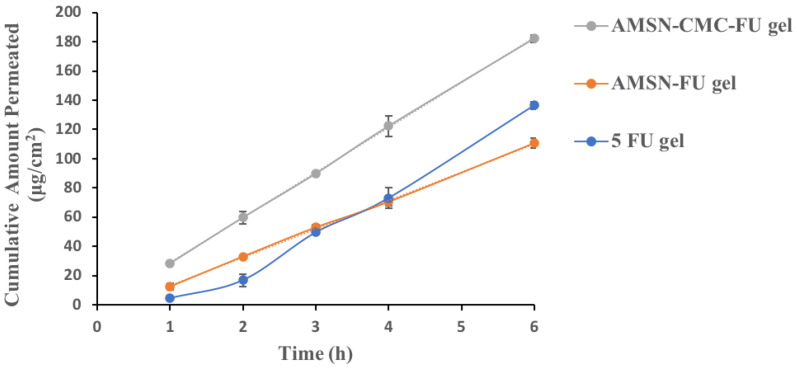
Ex vivo permeation of AMSN-FU gel, AMSN-CMC-FU gel, and 5-FU gel. The results are the mean of three measurements with standard deviation (Mean ± SD, *n* = 3).

The amount of 5-FU permeated per unit area per unit time (permeation-flux, J) and permeation coefficients (P*_app_*) were calculated for free 5-FU, 5-FU in AMSN, and 5-FU in AMSN-CMC (Table 2), and all formulations were used as Carbopol gel. The permeation profiles of the AMSN-FU and AMSN-CMC-FU gel formulations were different from that of free 5-FU gel. The nanoparticle gel formulation showed a linear permeation profile at all stages of the experiment, while the free 5-FU gel showed a linear profile at the later stage of the study [10]. The result showed that the permeation of 5-FU from the AMSN-CMC-FU was significantly (*p* < 0.01) increased compared to free 5-FU gel at all-time points (Figure 9). The permeability coefficient for AMSN-CMC-FU was higher than the free 5-FU gel and AMSN-FU gel, and the AMSN-CMC-FU formulation showed the shortest lag time. In addition, the AMSN-FU gel formulation showed higher permeation of 5-FU initially for up to 3 h (*p* < 0.05) than the 5-FU gel formulation. In the later stage after 3 h, the amount permeated of the drug was higher for the free 5-FU gel in comparison to AMSN-FU. Also, the lag time was shorter in AMSN-FU gel in comparison to free drug gel. The permeability coefficient for AMSN-FU gel was higher than the free 5-FU gel at the initial phase up to 2 h. The initial stage would be very important in this study as the integrity of the cell membrane could be affected by the cytotoxicity of the drug [10].

The drainage of precorneal fluid is one of the main reasons for the low ocular bioavailability of any drug. A large fraction of instilled dose (around 80–90%) drains into the nasolacrimal duct. The nasolacrimal drain maintains a precorneal fluid volume of about 7–10 μL [33]. Some factors can influence the bioavailability of a drug in the ocular delivery such as instilled volume, viscosity, pH, and the type of drug according to biopharmaceutical classification. The high viscosity of the instilled dose extends the precorneal retention and residence time of the dosage form and may improve drug ocular permeation. The physiological pH of tear fluid is 7.4 and the buffering capacity of tear fluid is very high so the slightly variable pH of the instilled dose will be buffered by the tear fluid to the ocular physiological pH. In addition, 5-FU is a weakly acidic drug with pKa of around 8 and 13, and a highly polar and weakly permeable drug (log P octanol/water = −0.89) [15,34]. Thus, with a proper formulation of this drug considering different physiological and formulation factors, the ocular bioavailability of 5-FU can be enhanced. All three formulations were applied as a gel to increase the viscosity of the applied dose, hence the residence time at the precorneal area. The pH was also adjusted to 7.4 using PBS to prevent irritation of the ocular tissues. Moreover, pH 7.4 would be suitable for 5-FU as not all fractions of the drug are in the ionized form where the ionized and nonionized fractions of 5-FU contribute to the overall membrane permeability of the drug [35]. Furthermore, the drug will be stable at this pH [36].

The nanoparticle gel delivery system is expected to enhance some drugs’ permeation and ocular bioavailability. Many researchers have proposed that the nanoparticles would facilitate transcorneal permeation through the cellular junctions due to enhanced precorneal retention [8,11,37]. Also, small particles with a mucoadhesive property would improve the bioavailability. Moreover, the controlled release of a drug from a nanocarrier system would further enhance the bioavailability as it maintains the concentration gradient at the corneal surface [32]. The results showed that the formulation of 5-FU in AMSN enhanced the ocular permeation, which could be due to controlling the release of the drug and also the mucoadhesion property of the AMSN. Additionally, coating AMSN with CMC further enhanced the permeability of the drugs. CMC on the surface of AMSN can act as a penetration enhancer since it can open the tight junctions located in the epithelial cells with mucoadhesion properties. According to numerous studies, coating nanoparticles with chitosan or chitosan derivatives can prolong the residence time in the cornea and enhance the intraocular penetration of drugs [8,11,37].

### 2.11. In Vivo Pharmacokinetic Study

**Figure 10:**
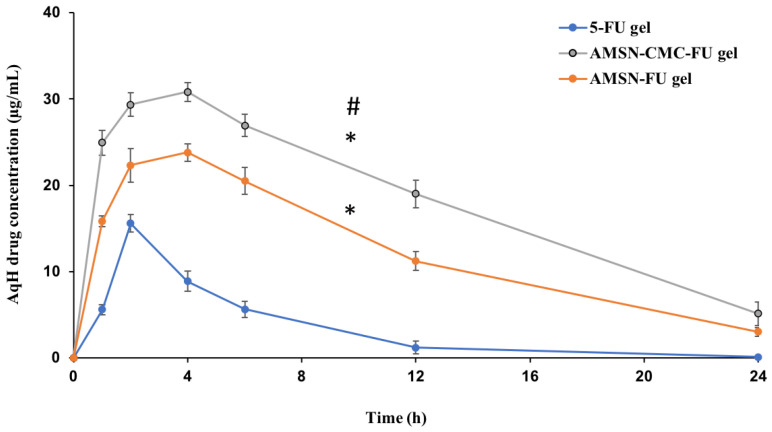
5-FU concentrations in AqH following topical ocular instillation of 5-FU gel, AMSN-FU gel, and AMSN-CMC-FU gel in rabbit eyes (mean ± SD, *n* = 3). * Represent the significant difference between the AMSN-CMC-FU gel or AMSN-FU gel parameters in comparison to the 5-FU gel. **#** Represent the significant difference between AMSN-CMC-FU gel and AMSN-FU gel.

A calibration curve of the UPLC-MS/MS method was successfully applied for 5-FU quantification in AqH of rabbit eyes after topical administration of 5-FU gel, AMSN-FU gel, and AMSN-CMC-FU gel. Typical chromatograms are presented in Figure S1 (Supplementary Materials). The detected concentrations of 5-FU in AqH samples extracted from rabbit eyes at 1, 2, 4, 6, 12, and 24 h are plotted against time as shown in Figure 10.

5-FU concentration in AqH in 5-FU gel formulation was lower than AMSN-CMC-FU and AMSN-FU gel formulations at all time points, which might be due to rapid corneal and precorneal loss of 5-FU in its gel form. The rate and extent of absorption and permeation of 5-FU from AMSN-CMC-FU gel, AMSN-FU gel, and the 5-FU gel were analyzed by comparing the pharmacokinetic parameters including C_max_, T_max_, t_1/2_, AUC, and MRT for the three treatment groups (Table 3).

The pharmacokinetic parameters were calculated by using PK-Solver software. The AqH 5-FU concentration-time plot was relatively smooth and suitable for carrying out an ocular pharmacokinetic data analysis of the formulations. By comparing the obtained pharmacokinetic data for the three formulations, AMSN-CMC-FU gel showed significantly higher ocular bioavailability of 5-FU than both AMSN-FU (*p* < 0.05) and 5-FU gel (*p* < 0.01). Noticeable, 1.52 and 6.14 times higher AUC_0-inf_ were observed with the AMSN-CMC-FU compared to AMSN-FU and 5-FU gel, respectively. The t_1/2_ of AMSN-CMC-FU was also significantly higher than that of the FU gel formulation with a 2.5 times increase in value (*p* < 0.05). A significant 2.3-fold increase in mean residence time (MRT_0-inf_) was also found for AMSN-CMC-FU gel compared to FU gel (*p* < 0.05). C_max_ and T_max_ also were higher for AMSN-CMC-FU gel compared to FU gel (*p* < 0.01). Conversely, AMSN-CMC-FU gel showed higher C_max_ in comparison to AMSN-FU gel (*p* < 0.01) but the same T_max_ at 4 h. Moreover, the t_1/2_ and MRT of AMSN-CMC-FU gel were higher than those of AMSN-FU gel but not to a statistically significant degree. The pharmacokinetic parameters C_max_, AUC_0-inf_, t_1/2_, and MRT of AMSN-FU gel were significantly higher than 5-FU gel (*p* < 0.01) with 1.5, 4, 2, and 2-fold increases in value, respectively. Furthermore, the clearance of the drug was significantly higher for 5-FU gel in comparison to AMSN-CMC-FU gel and AMSN-FU gel (*p* < 0.01), and the clearance for AMSN-FU gel was higher than that of the coated formulation gel (*p* < 0.01).

The elevated values of pharmacokinetic parameters in the case of nanoparticle-treated animals could be due to prolonged precorneal retention of the nanoparticles. 5-FU-loaded nanoparticle gel formulations were measured for up to 24 h in higher concentrations than the control gel formulation after ocular instillation. This indicates a fast pre-corneal loss of 5-FU when administered in free gel form. Prolonged release of 5-FU was shown by using chitosan nanoparticles, and controlling the release of the drug with a mucoadhesion system enhanced the ocular bioavailability of the drug [9]. Other studies involving other drugs loaded into nanoparticles for ocular delivery showed enhanced ocular bioavailability [7,38].

The positive zeta potential of AMSN could induce electrostatic attraction with the negatively charged mucin layer of the cornea and conjunctiva of the rabbit eyes [7]. Similar to other silica particles, there are many hydroxyl groups in the AMSN, allowing the formation of a hydrogen bond with the mucin. The amino groups of AMSN could adhere better to the mucin compared with mesoporous silica particles without amino groups, which could be due to the formation of an ionic complex of the amino group with the negatively charged mucin [7]. This electrostatic interaction would enhance ocular retention resulting in high transcorneal permeation of 5-FU compared to 5-FU gel.

Chitosan polymer possesses a mucoadhesion ability and can modulate the tight junctions of epithelial cells. Several studies showed that coated nanoparticles with chitosan or its derivatives would enhance the drug’s ocular bioavailability [8]. However, chitosan possesses low mucoadhesive strength at neutral pH and has some limitations such as low water solubility and low swelling properties. For example, the mucoadhesive strength of neat chitosan was estimated to be 0.34 N/cm, while Carbopol-934 had a much higher mucoadhesive strength of about 0.51 N/cm^2^. Several studies have modified chitosan with appropriate monomers to overcome these problems, which have additional reactive groups to ensure mucoadhesion at pH 7. This is very important for ocular release formulations since eye mucus has a pH of about 7.4 and thus can be characterized as a slightly basic surface [8]. The high aqueous miscibility and excellent physicochemical properties of CMC suggested that it may meet the requirement for increased ocular 5-FU permeability. CMC is an amphoteric derivative of chitosan, which, due to the presence of both carboxyl and amino groups, can act as an acidic or basic material, depending on the pH. These two functional groups would enhance the mucoadhesive strength of chitosan. CMC would also help in the paracellular diffusion of 5-FU as a modulator of tight cellular junction [8]. Moreover, the high biocompatibility, biodegradability, excellent antimicrobial activity, nontoxicity, and moisturizing capacity of CMC prove that it is an exceptional material for wound healing applications [31]. Overall, CMC would exert a protective effect on the corneal membrane layer membrane and enhance the absorption flux of 5-FU across the corneal barrier.

Nagarwal et al. evaluate the ocular delivery of 5-FU utilizing chitosan-coated sodium alginate–chitosan nanoparticles. They showed enhanced ocular bioavailability of 5-FU in comparison to the free drug solution with a dose of 200 μg of the drug [9]. Although they conducted their ocular pharmacokinetic study for up to 8 h, our AMSN formulations showed higher ocular bioavailability of the drug with a lower dose of 5-FU equivalent to 100 μg. Tissue culture studies have shown that the concentration of 5-FU that is required to induce an IC_50_ (drug concentration needed to inhibit cell proliferation by 50%) in rabbit conjunctival fibroblast proliferation is only 0.2 μg/mL [39]. It was also found in a study with rabbit eyes that after a single subconjunctival injection of 5-FU using a liposomal delivery system, an AqH concentration of 0.2 μg/mL was recorded for only 12 h. In our formulations of AMSN-CMC-FU gel and AMSN-FU gel, the 5-FU concentration in AqH would be maintained above 0.2 μg/mL for more than 24 h. Another study showed that the IC_50_ of 5-FU against uveal melanoma cell lines (OCM1, M23, and SP6.5) is around 2.6–0.4 μg/mL [40]. This concentration would be maintained in AqH by AMSN-CMC-FU gel and AMSN-FU gel formulation for longer than 24 h. In our previous work, we showed that AMSN-loaded 5-FU has superior activity in comparison to free 5-FU against a malignant human melanoma cell line (HT-144 cells) [15]. Therefore, the results suggest that the drug in AMSN gel formulations has the potential to be applied in a single daily dose with both prolonged release and enhanced bioavailability demonstrated over a 24 h period.

### 2.12. Ocular Irritation and Tolerability Study

The ocular irritation caused by the formulations 5-FU gel (control), AMSN-CMC-FU gel, and AMSN-FU compared to normal saline was examined for up to 24 h. Any inflammatory changes in the conjunctiva, cornea, and iris were inspected visually [41]. Based on the inflammatory signs and symptoms of eye irritation, the scoring was performed by grading and scoring [42] as summarized in Table S1 (Supplementary Materials). The pathological alterations include swelling, redness, chemosis, hemorrhage, mucoidal discharges (cloudiness), and edema [43]. The ocular irritation type was categorized [44] as mentioned in Table S2 and the obtained scores are summarized in Table 4.

**Figure 11:**
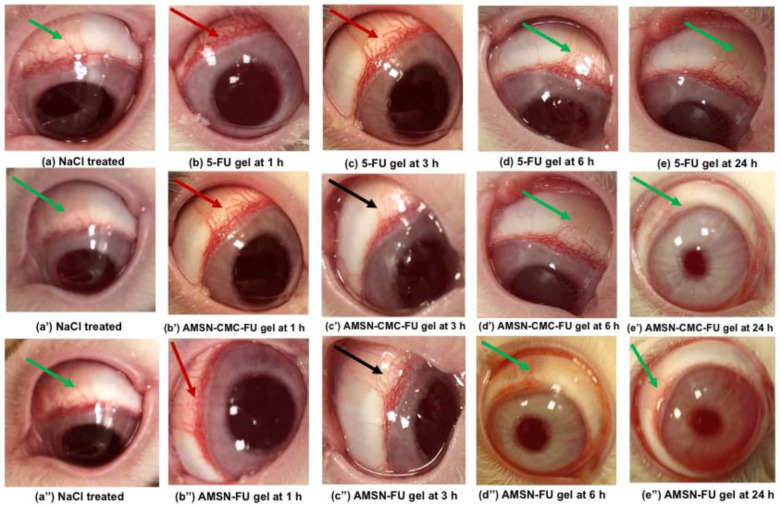
Eye images captured during irritation study. Representative images of normal saline-treated eyes (**a**,**a’**,**a”**) (green arrows). Post topical application of 5-FU gel at 1 h (**b**) (red arrow); at 3 h (**c**) (red arrow); at 6 h (**d**) (green arrow,) and at 24 h (**e**) (green arrow). Post application of 5-FU-AMSN-CMC at 1 h (**b’**) (red arrow); at 3 h (**c’**) (black arrow); at 6 h (**d’**) (green arrow), and at 24 h (**e’**) (green arrow). Post application of 5-FU-AMSN at 1 h (**b”**) (red arrow); at 3 h (**c”**) (black arrow); at 6 h (**d”**) (green arrow), and at 24 h (**e”**) (green arrow). Green arrow: no abnormality, red arrow: moderate’s uveitis, and black arrow: mild uveitis.

Normal features of eyes were observed in the treated rabbits during irritation testing of normal saline. Figure 11a,a’,a” are the representative images of normal saline-treated eyes. The 5-FU formulations treated animals exhibit inflammatory signs showing redness of the conjunctiva with mild mucoidal discharge (red arrow) 1 h post-application of 5-FU gel, AMSN-CMC-FU gel, and AMSN-FU gel (Figure 11b,b’,b”), respectively. The three treated rabbits with 5-FU gel exhibited moderate inflammatory signs and slight watery discharge even at 3 h (Figure 11c, red arrow), while AMSN-CMC-FU gel- and AMSN-FU gel-treated animals demonstrate mild inflammatory signs in the eyes at 3 h (Figure 11c’,c” black arrows). Mild inflammatory changes by these two formulations at 3 h were probably because of the limited or low toxicity of the 5-FU-containing formulations as well as attributed to the controlled release of the encapsulated drug. None of the treated rabbits showed inflammatory signs (such as redness of conjunctiva with mucoidal discharge) represented by green arrow 6 h and 24 h post administration of 5-FU gel, AMSN-CMC-FU gel, and AMSN-FU gel (Figure 11d,d’,d”) and (Figure 11e,e’,e”), respectively. The inflammatory changes in the conjunctiva, iris, and cornea disappeared and the treated eyes regained their normalcy 24 h post administration of 5-FU gel, AMSN-CMC-FU gel, and AMSN-FU gel [45].

The application of the 5-FU gel induced minimal irritation in one animal with slight redness and discharge, and it was given a score of 1. No corneal lesions were observed, thus the cornea, iris, and conjunctiva were given a score of 0. The maximum mean total score (MMTS) was calculated as per the irritation scoring system [44]. The MMTS for 5 FU gel, AMSN-CMC-FU gel, and AMSN-FU gel after 24 h of administration were 13.00 (>2.6 and <15.1, minimally) and 7.34 (>2.6 and <15.1, minimally) for both AMSN formulations, as shown in Table 5. Thus, the 5 FU gel, AMSN-CMC-FU gel, and AMSN-FU gel were “minimally irritating” to rabbit eyes. However, AMSN-CMC-FU and AMSN-FU had minimal eye irritation potency compared to the 5-FU gel.

## 3. Materials and Methods

### 3.1. Materials

N-Lauroylsarcosine sodium, 3-aminopropyltriethoxysilane (APTES), tetraethyl orthosilicate (TEOS), ammonium acetate, 5-fluorouracil, and dexamethasone were purchased from Sigma-Aldrich Chemical Co. (St Louis, MO, USA). Carbomer (Carbopol 934) was purchased from Acros Organics (Morris Plains, NJ, USA). O-Carboxymethyl chitosan (CMC) M. WT (100–300 KD) was purchased from Santacruz Biotechnology (Dallas, TX, USA). Mucin powder was supplied by Xian Kono Chem Co., Ltd. (Xi’an, China). All other chemical reagents used were of analytical grade and used without further purification.

### 3.2. Synthesis of Aminated Mesoporous Silica Nanoparticle (AMSN)

The preparation of AMSN was performed as previously described [15]. Briefly, N-Lauroylsarcosine sodium (1 mmol) was dissolved in 33 mL of a water:ethanol mixture (10:1), then 4 mL of 0.1 N HCl was added under stirring for 1 h. Then, 150 μL of APTES were added and stirred for 10 min. Thereafter, TEOS (1.5 mL) was added to the reaction mixture and stirred for 10 min. The suspension was sonicated using an ultrasonic water bath (Cole-Parmer SS, Cole-Parmer, Vernon Hills, IL, USA). Then, the suspension was left to rest for 1 h before being heated at 80 °C for 18 h. The nanoparticles were recovered by centrifugation at 10,000 rpm, washed with deionized water, and dried in an oven at 60 °C for 12 h. The surfactant was removed by solvent extraction where the obtained powder was dispersed in ammonium acetate (8.01 g) in 100 mL (4:1 ethanol: water) and refluxed at 90 °C for 12 h [15].

### 3.3. O-Carboxymethyl Chitosan (CMC) Coating of AMSN

An accurately weighed amount of CMC (40 mg) was dissolved in 20 mL of 0.5 M NaCl (2.0 mg/mL). Then 10 mg of AMSN was added to the CMC solution and stirred for 4 h at ambient temperature to obtain CMC-coated AMSN. The solution was centrifuged at 6000 rpm for 25 min and the unreacted CMC was removed by washing the particles with distilled water [26]. The recovered CMC-coated nanoparticles (AMSN-CMC) were lyophilized for 24 h for further characterization.

### 3.4. Loading of 5-FU into AMSN-CMC

To load 5-FU on AMSN-CMC, 5-FU was dissolved in 10 mL of PBS (pH 7.4) to make a 1 mg/mL solution. The AMSN-CMC (50 mg) was added to the previously prepared 5-FU solution in PBS. The suspension was sonicated for 10 min by probe sonication (Badnelin, Germany) and stirred for 24 h at room temperature. The evaporation of the solvent was prevented. The suspension was centrifuged at 6000 rpm for 1 h to separate the loaded AMSN-CMC-FU, then lyophilized for 24 h. The dried powder was used for further characterization. Loading 5-FU on AMSN was performed as described previously [15]. Briefly, to prepare 5-FU AMSN, 5-FU was dissolved in 25 mL of PBS (pH 7.4) to make a 1 mg/mL solution. Then, 25 mg of AMSN was added to the 5-FU in PBS solution, sonicated by probe sonication for 5 min at 30% power (Badnelin, Germany), and stirred at room temperature for 24 h. The suspension was centrifuged at 6000 rpm for 1 h to separate the loaded AMSN-FU, then lyophilized for 24 h. The supernatant samples were collected to calculate the Encapsulation efficiency (EE%) and loading capacity (LC%). EE% and LC% were determined by measuring the drug concentration in the supernatant. LC% was further confirmed and calculated by extracting the loaded drugs by the following procedure. The loaded drugs in AMSN-CMC or AMSN were extracted using PBS (pH 7.4). Accurately weighed nanoparticle (5 mg) was dispersed in PBS and sonicated for 15 min using probe sonication at 80% power (Badnelin, Germany). Then, the nanoparticles suspension was shaken in the water bath for 3 days. The amount of drug loaded in the nanoparticles was determined using HPLC (Agilent, Santa Clara, CA, USA) for 5-FU [46].

*EE*% and *LC%* were calculated according to the following equations:

(1) $$EE\%=\frac{\left(W_{total}\;-W_{Free}\right)\times 100}{W_{total}\;\;\;\;}$$

(2) $$LC\%=\frac{\left(W_{total}\;-W_{Free}\right)\times 100}{W\;\;\;\;}\;$$

where *W_total_* is the initial weight of the drug before loading, *W_free_* is the excess amount of drug in the solution, and *W* is the total weight of the drug and AMSN [15].

### 3.5. Preparation of Gel Formulations

Carbopol 934 gel was selected as hydrogel according to the previous work, which provides good flow properties of nanoparticles for the topical applications field [47]. Carbopol 934 was dispersed in PBS to prepare 1% *w*/*v*. The mixture was stirred at room temperature for 3 h and the pH was adjusted to 7.4 using triethanolamine (0.5%, *w*/*v*). The gel was allowed to stand overnight to remove any trapped air. An appropriate amount of 5-FU or loaded into AMSN or AMSN-CMC was inserted into the gel to prepare a final concentration of 0.25% *w*/*w* for 5-FU and the formulations were stirred at room temperature for 24 h to mix uniformly without any aggregation until the gels were homogeneous. All formulated gels were described for their cosmetic characteristics (color, texture, and consistency). The gels were light yellowish with a smooth homogeneous appearance and texture. The pH value of the formulations was adjusted to 7.4 using 0.1 N NaOH.

### 3.6. Particle Morphology

#### 3.6.1. Transmission Electron Microscope (TEM)

The morphology of the AMSN-CMC-FU formulation was determined by transmission electron microscope (TEM) (JEM-1011, JEOL, Tokyo, Japan) at 60 kV.

#### 3.6.2. Scanning Electron Microscopy (SEM)

The shape and surface characteristics of the AMSN-CMC-FU formulation were observed by scanning electron microscopy (SEM) (Zeiss EVO LS10; Cambridge, UK and FESEM (JSM-7600F, JEOL Inc., Akishima, Japan)) using the gold sputter technique. The formulation was vacuum dried and coated with gold in a Q150R Sputter unit from Quorum Technologies Ltd. (East Sussex, UK) for 60 s in an argon atmosphere at 20 mA. The zone magnification for the images was kept at around 10,000–15,000x. Observations were performed under 1 and 15 kV.

### 3.7. Particle Size, Polydispersity, and Zeta Potential Measurements

The particle size, polydispersity index (PDI), and zeta-potential of the nanoparticles were measured by a Zetasizer Nano series-ZS (Malvern Instruments, Malvern, UK) at a concentration of 100 μg/mL. The dynamic light scattering (DLS) technique was used to evaluate the particle size and size distribution (PDI) of nanoparticles at 25 °C. Laser Doppler Velocimetry (LDV) mode of the same instrument was used to measure the zeta potential (mV) of the nanoparticles after an appropriate dilution with distilled water at 25 °C. All experiments were performed in triplicate.

### 3.8. Proton Nuclear Magnetic Resonance (^1^H-NMR)

^1^H-NMR spectra were obtained using a Bruker Ultra shield 500.133 MHz spectrometer. Baseline correction, calibration, and processing were performed using Topspin software. The samples of AMSN, CMC, and the conjugate (AMSN-CMC) were dissolved in a sodium hydroxide (100 mg/mL) in D_2_O and measured at a concentration of 20 mg/mL. Chemical structures were shown by using the CS ChemDraw^®^ program for illustration.

### 3.9. Thermogravimetric Analyses (TGA)

TGA analyses were performed using a differential scanning calorimeter (TGA-DSC; SDT Q600 V20.9 Build 20, TA Instruments, Champaign, IL, USA) at a heating rate of 5 °C/min from room temperature to 700 °C under a nitrogen flow of 50 mL/min.

### 3.10. Powder X-ray Diffraction (PXRD)

Diffraction patterns were evaluated using an Ultima-IV diffractometer (Rigaku, Tokyo, Japan) over a 2θ range of 3 to 60° at the rate of 0.5 degree/min scan speed. The tube anode was Cu with Ka = 0.1540562 nm monochromatized with a graphite crystal. The patterns were collected at a tube voltage of 40 kV and tube current of 40 mA in step scan mode (step size 0.02°, counting time 1 s per step).

### 3.11. Fourier Transform Infrared Spectroscopy (FTIR)

FTIR (Nicolet Magna-IR 550 FTIR spectrometer, range of 4000–500 cm^−1^) was used to characterize the structural composition of the samples. The FTIR spectra were recorded in the diffuse reflectance mode (Nicolet 60SXB).

### 3.12. Evaluation of the Mucoadhesive Strength Using Mucin (Particle Method)

The particle method is a simple technique that depends on evaluating the zeta potential of a mucin suspension before and after adding a known amount of the particles. Briefly, a mucin powder suspension (0.1 mg/mL) was prepared by adding the calculated amount of bovine mucin into a 100 mM phosphate buffer solution pH (7.4) and mixed overnight to ensure complete dispersion. A known weight (10 mg) from each of the nanoparticles was dipped into a test tube filled with 3 mL of the mucin suspension, and the suspension was kept water bath for 48 h with continuous shaking. Zeta potentials of the prepared suspensions and the mucin powder suspension were recorded using a Zetasizer Nano series-ZS (Malvern Instruments, UK) [27,28].

### 3.13. In Vitro Drug Release

To facilitate a localized easy ocular application, Carbopol was used as a topical vehicle for 5-FU-loaded AMSN (AMSN-FU) and 5-FU-loaded AMSN-CMC (AMSN-CMC-FU) and compared for free drug gel. A dialysis membrane molecular weight cut-off of 12000–14,000 (Sigma Co., St. Louis, MO, USA) was used. The membrane opening was tied to the mouth of a polyvinyl test tube and dipped in a beaker containing PBS (pH 7.4, 12 mL). The release of AMSN-FU and AMSN-CMC-FU was performed using modified Vertical Franz diffusion cells. The receptor phase consisted of 12 mL of PBS (pH 7.4) solution and was maintained at 37 ± 1 °C with constant stirring. 1 mL of 0.25% *w*/*v* 5-FU (equivalent to 2.5 mg) free drug gel, AMSN-FU, or AMSN-CMC-FU nanoparticle gel formulations was applied to the dialysis membrane. The donor part was exposed to PBS for 24 h. At predetermined time intervals, 1 mL of the receiver part (release media) was withdrawn, and an equivalent volume of PBS was added to the release media to maintain sink condition. 5-FU quantities were analyzed in the samples using HPLC [9,11].

### 3.14. Ex Vivo Corneal Permeation

An ex vivo permeation study was performed on excised goat eye. The freshly excised cornea was fixed in a Franz diffusion cell with the epithelial surface of the cornea facing the donor part. The receptor part was filled with phosphate buffer (pH 7.4) while 1 mL of 0.25% *w*/*v* of 5-FU (equivalent to 2.5 mg) gel or nanoparticle gel formulations was placed in the donor part. The corneal surface area was exposed to the donor cell and made available for drug permeation. The buffer solution was preheated and maintained during a study by the continuous flow of water in the outer jacketing of the receptor chamber at 37 ± 1 °C. The magnetic beads were placed in the receptor chamber and stirred at 100 rpm. Samples (0.5 mL) were withdrawn from the receptor cell at a predetermined time of up to 6 h and analyzed by HPLC [9]. The corneal permeation area through the Franz diffusion cell was 1.7 cm^2^, and the volume of the release medium in the receptor compartment was 12 mL. The amount of 5-FU permeated per unit area per unit time (permeation-flux, J) and the apparent permeability coefficient (*P_app_*) were calculated for free 5-FU, 5-FU in AMSN, and 5-FU in AMSN-CMC. The permeated amount of the drug (μg/cm^2^) through the cornea was calculated by considering the volume of the receptor compartment (12 mL), *DF* is the dilution factor, the involved corneal cross-section area, and the drug concentration (*conc*.) using Equation (3).

(3) $$Permeated\;amount\;of\;the\;drug\;\left(\mu g/{cm}^{2}\right)=\frac{Conc.\left(\mu g/mL\right)\times DF\times Volume\;of\;the\;receptor\;\left(mL\right)}{Area\;of\;the\;cornea\;\left({cm}^{2}\right)}$$

The flux (*J*) across the cornea was estimated from linear ascents of the permeation plot by the following equation:

(4) $$J\;\left(\mu g/cm\cdot h^{-1}\right)=\frac{dQ}{dt}\;$$

where ‘*Q*’ is the amount of drug crossed through the cornea or *dQ/dt* is the linear portion of the slope and ‘*t*’ is the time of exposure.

The apparent permeability (*P_app_*) was estimated by the following equation:

(5) $$P_{app}\;\left(cm/h\right)=\frac{J}{Co}$$

where *C_0_* is the initial drug concentration (2500 μg/mL) in the donor part [12].

### 3.15. In Vivo Pharmacokinetic (PK) Studies

In vivo experiments were performed on groups of three albino rabbits model weighing from 3 to 4 kg, free of any signs of abnormality. Each conscious animal received 40 μL from 0.25% of 5-FU (equivalent to 100 μg 5-FU) from AMSN-CMC-FU in Carbopol gel or AMSN-FU in Carbopol gel of sterile suitable concentration in the cul-de-sac of the right eye or free 5-FU gel. Normal saline was instilled in the left eye and used as a control. One-hour post instillation, the animals were sedated with an intra-muscular injection of a mixture of Ketamine, HCl and Xylazine (15 and 3 mg/kg of body weight of each, respectively). One drop of Proparacaine.HCI (0.5%, *w*/*v*) and one drop of Tropicamide (1%, *w*/*v*) were put into the treated eyes to enlarge the pupil. Thereafter, 40–50 μL of Aqueous humor (AqH) samples were taken out by using a 29-Gauge needle attached to a syringe at different time points. AqH was collected at predetermined time points (1, 2, 4, 6, 12, and 24 h) from the treated eyes of all the animals of respective groups and stored at −80 °C until analysis. Ethyl acetate was added to samples to precipitate the protein and separated by cooling centrifuge at 10,000 rpm for 10 min. The supernatant was collected and evaporated. Finally, the sample was reconstituted in acetonitrile and analyzed by UPLC-MS/MS for 5-FU using allopurinol as the internal standard. All PK parameters were resolved by the non-compartmental PK analysis method (PK-Solver, Nanjing, China in MS-Excel-2013). The concentration of 5-FU in AqH was measured from the relative recovery of the drug in the aspirated AqH samples.

### 3.16. UPLC-MS/MS Analysis of 5-FU

#### 3.16.1. Chromatographic and Mass Spectrometric Conditions

A previously reported ultra-performance hydrophilic interaction liquid chromatography coupled with tandem mass spectrometry method was used for quantification of the 5-FU in AqH with slight modification [48]. Briefly, an Acquity™ UPLC system connected with a triple-quadruple tandem mass spectrometer detector (TQD) was used (Waters Corp., Milford, MA, USA). Allopurinol was used as internal standard (IS). The chromatographic separation of 5-FU and IS was achieved on an Acquity UPLC BEH HILIC column (2.1 × 100 mm, 1.7 μm) fitted with a 0.2 μm stainless steel frit filter (Waters Corp., Milford, MA, USA) and maintained at a constant 40 °C temperature. The mobile phase consisted of acetonitrile:10 mM ammonium acetate (95:5, *v*/*v*). The flow rate was 0.3 mL/min, the volume of injection was 5 μL, and the total run time was 2.5 min. The 5-FU and IS were separated between 1 and 2 min with retention times of 1.13 and 1.47 min, respectively. A TQD equipped with an electrospray ionization interface was operated in a negative mode to detect 5-FU and IS. The optimized TQD parameters were: 150 °C (source temperature), 0.93 kV (capillary voltage), 0.245 s (dwell time), 350 °C (desolvation temperature), 600 L/h (desolvation gas flow rate), 50 L/h (cone gas flow rate) and 0.2 mL/min (argon as collision gas flow rate). The optimized MS/MS conditions were: cone voltage 32 V for 5-FU and 40 V for IS, and the collision energy was 14 eV for 5-FU and 20 eV for IS. Multiple reactions monitoring (MRM) was used to quantify the 5-FU and IS with parent-to-daughter ion transitions (m/z) of 128.92→41.98 and 134.94→64.1, respectively. N2 was used as desolvation and cone gas while argon was the collision gas. The UPLC-MS/MS system was operated by Mass Lynx software (version 4.1, SCN 714), and data was collected and processed using the LynxTM program [48].

#### 3.16.2. Sample Preparation in Aqueous Humor (AqH)

AqH (25 μL) samples were added to 250 μL of the IS working solution (1 μg/mL of allopurinol in ethyl acetate) and mixed in Eppendorf tubes and gently vortexed for 1 min. After vortex-mixing, all samples were centrifuged at 13,500 rpm for 10 min. The samples were transferred for cold centrifugation (4 °C) at 13,500 rpm for 10 min. Then, the separated organic layer was transferred into a fresh tube and dried in a sample concentrator maintained at medium temperature (Thermo Scientific, Waltham, MA, USA). Then, the dried samples were reconstituted in 75 μL of acetonitrile and transferred to UPLC vials. Finally, 5 μL of the reconstituted solutions were injected into the UPLC-MS/MS system for 5-FU quantification.

### 3.17. Ocular Irritation and Tolerability Study

This study was performed in healthy rabbits by following Draize’s test [49]. We followed the guidelines of “The Association for Research in Vision and Ophthalmology (ARVO)” for animal use in “Ophthalmic and Vision Research”. Thus, the right eyes were selected for the products to be tested and the left eyes were treated with normal saline (0.9% NaCl). Generally, six rabbits are taken for one product to be tested in the eyes. In the present investigation, we used three animals for one product, and it was expected that there was a small chance of severe eye irritation and damage due to the presence of AMSN in the products. Additionally, only a limited number of rabbits were available for use. Nine rabbits were divided into three groups, three for 5-FU gel, three for AMSN-CMC-FU gel, and three for AMSN-FU gel. For acute eye irritation, at 10–15 min intervals three consecutive doses of 5 FU-containing products (40 μL) were administered in the right eyes of each rabbit. After 1 h of dosing, the eyes were periodically observed visually for up to 24 h for any signs and symptoms in the cornea, conjunctiva, and iris or any alteration in the treated eyes compared to the left eyes (treated with normal saline). Pictures of the eyes were captured for scoring purposes and the level of irritation was assessed based on animal discomfort, the signs, and symptoms such as redness, swelling, chemosis in the conjunctiva, iris, and cornea, or any mucoidal or watery or non-mucoidal discharges [38,42]. The irritation scoring was performed and the irritation due to the tested products was categorized following the previously reported literature [43,44]. Table S1 and S2 illustrated the grading and scoring systems that were followed for the obtained score for each formulation.

### 3.18. Statistical Analysis

The results were articulated as the mean of three measurements with standard deviations (Mean ± SD, *n* =3) and analyzed for statistical significance (*p* < 0.05) by Paired t-test (GraphPad Software Inc., San Diego, CA, USA).

## 4. Conclusions

The optimized AMSN-CMC-FU gel we developed was deemed suitable for ocular drug delivery of 5-FU. The developed nanosystem is a promising formulation to deliver 5-FU safely and efficiently to the eye. Around 2.48- and 2-times increases in t_1/2_ and C_max_ were found for AMSN-CMC-FU gel in comparison to 5-FU gel. respectively. In addition, 6.14-, 13.16-, and 2.27-fold increases in AUC_0-inf_, AUMC_0-inf_, and MRT_0-inf_ were found for AMSN-CMC-FU gel in comparison to 5-FU gel, respectively. The clearance of 5-FU was slower (0.20 ± 0.02 mL/h) from AMSN-CMC-FU gel in comparison to 5-FU gel (1.26 ± 0.18 mL/h). The CMC-coated formulation (AMSN-CMC-FU gel) showed an increase in transcorneal permeation of 5-FU in the ex vivo experiment, in good agreement with the pharmacokinetic study, which showed an improvement in ocular bioavailability of the drug in vivo. Moreover, the uncoated formulation AMSN-FU gel is a promising alternative for the ocular delivery of 5-FU, which showed lower pharmacokinetic performance in comparison to the CMC-coated formulation. The clearance of 5-FU was faster from 5-FU gel compared to the nanoparticle gel formulations. Relatively, an extended half-life of 5-FU and prolonged ocular retention from nanoparticle gel was found in comparison to the 5-FU gel formulation. The ocular irritation study indicated that the reference formulation 5-FU gel was “minimally irritating” while the AMSN formulations had minimal eye irritation potency compared to the 5-FU gel. Overall, the AMSN gel formulations would be promising for their ocular application. Furthermore, in vivo studies with animal cancer models could be investigated to check the anticancer efficacy of 5-FU such as uveal melanoma, including the different ocular anterior and posterior segment malignancy conditions following the topical application of the developed formulations. Future work will capitalize on the developed nanocarriers to enhance their properties as an optimal ocular drug delivery system for the treatment of ocular cancer.

## Figures and Tables

**Table 1:** Encapsulation efficiency (EE%) and drug loading (LC%) of AMSN-FU, AMSN-CMC-FU (F1), and AMSN-CMC-FU (F2). Results are the mean of three measurements with standard deviations (Mean ± SD, *n* = 3).

Formulations	EE%	LC%
AMSN-FU	18 ± 3.7	15.3 ± 3.1
AMSN-CMC-FU (F1)	10.3 ± 3.3	1.9 ± 0.6
AMSN-CMC-FU (F2)	25.8 ± 5.8	5.2 ± 1.2

**Table 2:** Parameters of transcorneal permeation for AMSN-FU gel, AMSN-CMC-FU gel, and 5-FU gel. The values are represented as the mean of three measurements with standard deviation (Mean ± SD, *n* = 3).

Formulations	Flux (J) (μg·cm^2^h^−1^)	Lag Time (h)	Cumulative Amount Permeated After 6 h (μg·cm^−2^)	Permeability Coefficient (cm·h^−1^)
AMSN-FU gel	19.4 ± 0.8	0.3 ± 0.087	110.5 ± 3.4	7.8 × 10^−3^ ± 2.9 × 10^−4^
AMSN-CMC-FU gel	30.8 ± 2.4	0.1 ± 0.03	182.2 ± 11.4	12.3 × 10^−3^ ± 1 × 10^−3^
5-FU gel (initial rate)	12.5 ± 2.5	0.6± 0.03	-	5 × 10^−3^ ± 9 × 10^−4^
5-FU gel (overall all permeation rate)	23.6 ± 0.1	0.7 ± 0.06	136 ± 2.5	9.4 × 10^−3^ ± 3 × 10^−4^

**Table 3:** Ocular pharmacokinetic (PK) parameters of 5-FU gel, AMSN-CMC-FU gel, and AMSN-FU gel (mean ± SD, *n* = 3). * Represent the significant difference between the AMSN-CMC-FU gel or AMSN-FU gel parameters in comparison to the 5-FU gel. **#** Represent the significant difference between AMSN-CMC-FU gel and AMSN-FU gel.

Pharmacokinetic Parameters	Formulations		
	5-FU gel	AMSN-CMC-FU gel	AMSN-FU
	Mean ± SD	Mean ± SD	Mean ± SD
t_1/2_ (h)	3.0 ± 0.2	7.6 ± 1.4 *	6.6 ± 0.5 *
T_max_ (h)	2.0 ± 0.0	4.0 ± 0.0 *	4.0 ± 0.0 *
C_max_ (μg/mL)	15.6 ± 1.0	30.8 ± 1.1 *#	23.8 ± 1.0 *
AUC_0–24 h_ (μg/mL·h^−1^)	80.6± 13.3	440.1 ± 21.1 *#	298 ± 4.0 *
AUC_0-inf_ (μg/mL·h^−1^)	81.0 ± 13.3	497.6 ± 44.2 *#	327.1 ± 8.5 *
AUMC_0-inf_ (μg/mL·h^−2^)	413.9 ± 105.9	5835.7 ± 1292.1 *#	3393.2 ± 304.1 *
MRT_0-inf_ (h)	5.1 ± 0.5	11.6 ± 1.6 *	10.4 ± 0.7 *
Vz/F (mL)	5.5 ± 1.0 *	2.2 ± 0.2	2.9 ± 0.1 #
Cl/F (mL/h)	1.3 ± 0.2 *	0.2 ± 0.02	0.31 ± 0.01 #

**Table 4:** Weighted scores for the eye irritation test of 5 FU-containing formulations.

Lesions in the Treated Eyes	Individual Scores for Eye Irritation by					
	5 FU Gel			Normal Saline (0.9% NaCl)		
	In Rabbit Number			In Rabbit Number		
	1st	2nd	3rd	1st	2nd	3rd
Cornea						
a. Opacity	1	0	0	0	0	0
b. Involved area of cornea	4	4	4	4	4	4
Total scores = (a × b × 5) =	20	0	0	0	0	0
Iris						
a. Lesion values	1	0	0	0	0	0
Total scores = (a × 5) =	5	0	0	0	0	0
Conjunctiva						
a. Redness	1	0	0	0	0	0
b. Chemosis	0	0	0	0	0	0
c. Mucoidal discharge	1	0	0	0	0	0
Total scores = (a + b + c) × 2 =	4	0	0	0	0	0
	AMSN-CMC-FU gel			Normal saline (0.9% NaCl)		
Cornea						
a. Opacity	0	0	1	0	0	0
b. Involved area of cornea	4	4	4	4	4	4
Total scores = (a × b × 5) =	0	0	20	0	0	0
Iris						
a. Lesion values	0	0	0	0	0	0
Total scores = (a × 5) =	0	0	0	0	0	0
Conjunctiva						
a. Redness	0	0	1	0	0	0
b. Chemosis	0	0	0	0	0	0
c. Mucoidal discharge	0	0	0	0	0	0
Total scores = (a + b + c) × 2 =	0	0	2	0	0	0
	AMSN-FU gel			Normal saline (0.9% NaCl)		
Cornea						
a. Opacity	0	1	0	0	0	0
b. Involved area of cornea	4	4	4	4	4	4
Total scores = (a × b × 5) =	0	20	0	0	0	0
Iris						
a. Lesion values	0	0	0	0	0	0
Total scores = (a × 5) =	0	0	0	0	0	0
Conjunctiva						
a. Redness	0	1	0	0	0	0
b. Chemosis	0	0	0	0	0	0
c. Mucoidal discharge	0	0	0	0	0	0
Total scores = (a + b + c) × 2 =	0	2	0	0	0	0

**Table 5:** Calculation of Maximum Mean Total Score (MMTS) by considering the obtained scores.

5-FU Gel					
In rabbit	1st	2nd	3rd	SUM	Average (SUM/3)
Cornea	0	0	20	20	6.67
Iris	5	0	0	0	5.00
Conjunctiva	0	0	4	4	1.33
SUM total =	5	0	24	24	13.00
**AMSN-CMC-FU gel**					
**In rabbit**	**1st**	**2nd**	**3rd**	**SUM**	**Average (SUM/3)**
Cornea	0	0	20	20	6.67
Iris	0	0	0	0	0.00
Conjunctiva	0	0	2	2	0.67
SUM total =	0	0	22	22	7.34
**AMSN-FU gel**					
**In rabbit**	**1st**	**2nd**	**3rd**	**SUM**	**Average (SUM/3)**
Cornea	0	20	0	20	6.67
Iris	0	0	0	0	0.00
Conjunctiva	0	2	0	2	0.67
SUM total =	0	22	0	22	7.34

## Data Availability

Data are available upon request from the corresponding author.

## Supplementary Materials

The following supporting information can be downloaded at: https://www.mdpi.com/article/10.3390/molecules28031260/s1, Table S1: Grading system for ocular irritation test; Table S2: Classification of eye irritation scoring system; Figure S1: Typical chromatograms of the LLOQ, blank AqH, and representative extracted sample from the ocular pharmacokinetic experiment.
