# Supplementary material for: Mesoporous Silica Nanoparticles Coated with Carboxymethyl Chitosan for 5-Fluorouracil Ocular Delivery: Characterization, In Vitro and In Vivo Studies
Source: Molecules. 2023 Jan 27;28(3):1260. doi: 10.3390/molecules28031260 (PMC9920178; doi:10.3390/molecules28031260)
Supplement: Supplementary file 1 [file molecules-28-01260-s001.zip › molecules-2144169-supplementary.pdf]

Mesoporous Silica Nanoparticles Coated with Carboxymethyl Chitosan for 5-Fluorouracil Ocular Delivery: Characterization, In Vitro and In Vivo Studies

Adel Ali Alhowyan [1], Mohd Abul Kalam [1,2], Muzaffar Iqbal [3,4], Mohammad Raish [1], Ahmed M. El-Toni [5,6], Musaed Alkholief [1,2], Aliyah A. Almomen [2,3] and Aws Alshamsan [1,2,*]

[1] Department of Pharmaceutics, College of Pharmacy, King Saud University, Riyadh 11451, Saudi Arabia
[2] Nanobiotechnology Unit, College of Pharmacy, King Saud University, Riyadh 11451, Saudi Arabia
[3] Department of Pharmaceutical Chemistry, College of Pharmacy, King Saud University, Riyadh 11495, Saudi Arabia
[4] Central Lab, College of Pharmacy, King Saud University, Riyadh 11451, Saudi Arabia
[5] King Abdullah Institute for Nanotechnology, King Saud University, Riyadh 11495, Saudi Arabia
[6] Nanomaterials and Nanotechnology Department, Central Metallurgical Research and Development Institute (CMRDI), Helwan, Cairo 11865, Egypt
* Correspondence: aalshamsan@ksu.edu.sa

Table S1. Grading system for ocular irritation test.

Cornea		
**Lesion**		**Score**
**a. Opacity-Degree of density (area which is most dense is taken for reading)**		
No ulceration or opacity		**0**
Scattered or diffuse area – details of iris clearly visible		**1**
Easily discernible translucent areas, details of iris slightly obscured		**2**
Opalescent areas, no details of iris visible, size of pupil barely discernible		**3**
Opaque, iris invisible		**4**
**b. Area of cornea involved**		
One quarter (or less) but not zero		**1**
Greater than one quarter but less than one half		**2**
Greater than one half but less than three quarters		**3**
Greater than three quarters up to whole area		**4**
**Score equals (a x b x 5):**	**Total maximum =**	**80**
**Iris**		
**Lesion**		**Score**
**a. Values**		
Normal		**0**
Folds above normal, congestion, swelling, circumcorneal injection (any one or all of these or combination of any thereof), iris still reacting to light (sluggish reaction is positive)		**1**
No reaction to light, hemorrhage; gross destruction (any one/ all of these)		**2**
**Score equals (a x 5):**	**Total maximum =**	**10**
**Conjunctiva**		
**Lesion**		**Score**
**a. Redness (refers to palpebral conjunctiva only)**		
Vessels normal		**0**
Vessels definitely injected above normal		**1**
More diffuse, deeper crimson red, individual vessels not easily discernible		**2**
Diffuse beefy red		**3**
**b. Chemosis**		
No swelling		**0**
Any swelling above normal (includes nictitating membrane)		**1**

Obvious swelling with partial eversion of the lids	**2**
Swelling with lids about half closed	**3**
Swelling with lids about half closed to completely closed	**4**
**c. Discharge**	
No any discharge	**0**
Any amount different from normal (does not include small amount observed in inner canthus of normal animals)	**1**
Discharge with moistening of the lids and hairs just adjacent to the lids	**2**
Discharge with moistening of the lids and considerable area around the eye	**3**
**Score equals (a + b + c) x 2: Total maximum =**	**20**

Note: The maximum total score is the sum of all scores obtained for the cornea, iris and conjunctiva. Scores of 0 are assigned for each parameter if the cornea, iris, or conjunctiva is normal.

Table S2. Classification of eye irritation scoring system.

Classification of Irritation	Maximum Mean Total Score (MMTS)
None	0.0-0.5
Practically none	0.6-2.5
Minimally	2.6-15.0
Mildly	15.1-25.0
Moderately	25.1-50.0
Severely	50.1-80.0
Extremely	80.1-100.0
Maximally	100.1-110.0

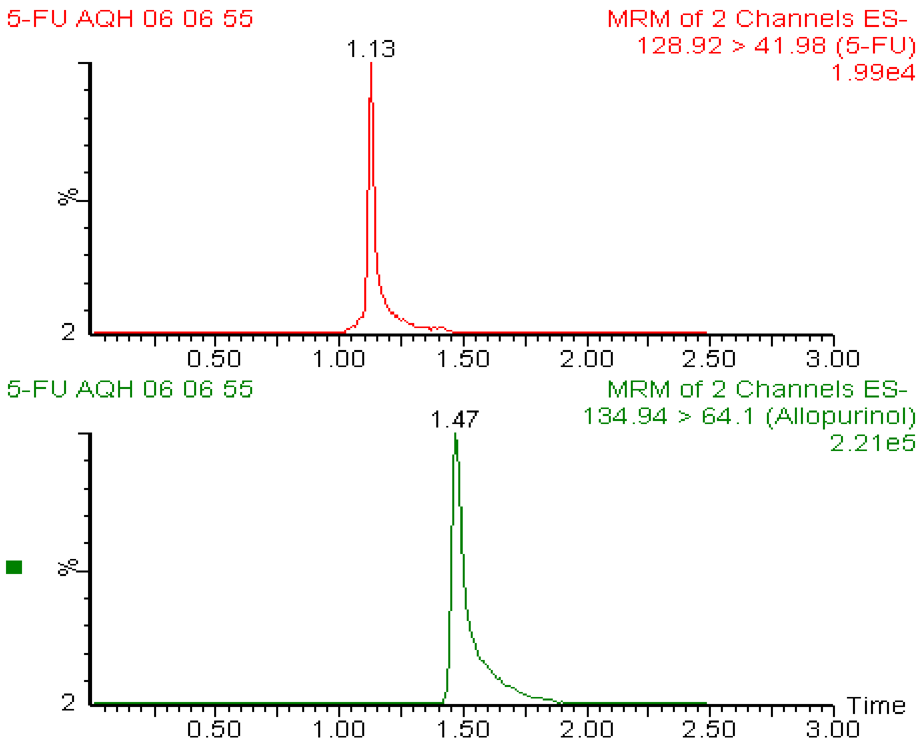

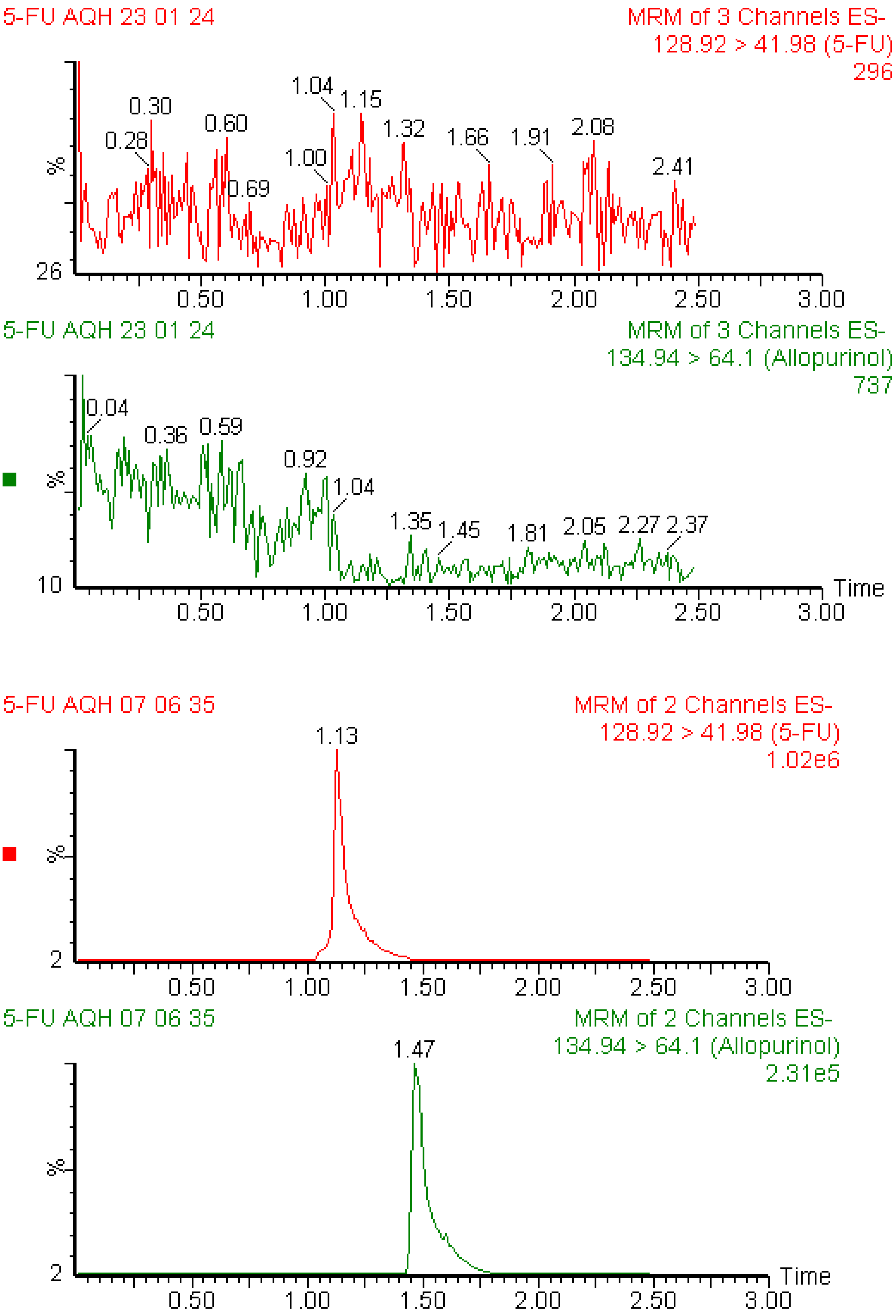

Figure S1. Typical chromatogram of LLOQ, blank aqueous humor, and representative extracted sample from ocular pK experiment.
